# The Role of Epigenetic Functionalization of Implants and Biomaterials in Osseointegration and Bone Regeneration—A Review †

**DOI:** 10.3390/molecules25245879

**Published:** 2020-12-12

**Authors:** Farah Asa’ad, Goda Pelanyte, Jincy Philip, Christer Dahlin, Lena Larsson

**Affiliations:** 1 Department of Biomaterials, Institute of Clinical Sciences, Sahlgrenska Academy, University of Gothenburg, P.O. Box 412, SE 405 30 Göteborg, Sweden; jincy.philip@gu.se (J.P.); christer.dahlin@biomaterials.gu.se (C.D.); 2 Institute of Odontology, University of Gothenburg, SE 405 30 Göteborg, Sweden; guspelgo@student.gu.se; 3 Department of ENT/Oral & Maxillofacial Surgery, NU-Hospital Organization, SE 461 85 Trollhättan, Sweden; 4 Department of Periodontology, Institute of Odontology, University of Gothenburg, SE 405 30 Göteborg, Sweden; lena.larsson@odontologi.gu.se

**Keywords:** epigenomics, biomedical and dental materials, dental implants, microRNAs, bone regeneration, osseointegration

## Abstract

The contribution of epigenetic mechanisms as a potential treatment model has been observed in cancer and autoimmune/inflammatory diseases. This review aims to put forward the epigenetic mechanisms as a promising strategy in implant surface functionalization and modification of biomaterials, to promote better osseointegration and bone regeneration, and could be applicable for alveolar bone regeneration and osseointegration in the future. Materials and Methods: Electronic and manual searches of the literature in PubMed, MEDLINE, and EMBASE were conducted, using a specific search strategy limited to publications in the last 5 years to identify preclinical studies in order to address the following focused questions: (i) Which, if any, are the epigenetic mechanisms used to functionalize implant surfaces to achieve better osseointegration? (ii) Which, if any, are the epigenetic mechanisms used to functionalize biomaterials to achieve better bone regeneration? Results: Findings from several studies have emphasized the role of miRNAs in functionalizing implants surfaces and biomaterials to promote osseointegration and bone regeneration, respectively. However, there are scarce data on the role of DNA methylation and histone modifications for these specific applications, despite being commonly applied in cancer research. Conclusions: Studies over the past few years have demonstrated that biomaterials are immunomodulatory rather than inert materials. In this context, epigenetics can act as next generation of advanced treatment tools for future regenerative techniques. Yet, there is a need to evaluate the efficacy/cost effectiveness of these techniques in comparison to current standards of care.

## 1. Introduction

### 1.1. Pitfalls of Current Biomaterials/Implants

Implant biomaterials intended for permanent anchorage within the alveolar bone typically include metals and alloys [1]. Bulk metallic glasses represent a more recent development, and a wide range of compositions exist, including Au-, Co-, Fe- Mg-, Ti-, Pd-, Pt, Zr-based systems [2]. The design features of an implant (including surface and bulk characteristics) play a key role in the early/initial osteogenic response, and indeed a range of surface modification methods have been pursued over the years [3,4]. Many of these are likely to directly influence peri-implant bone quality by modulating the attachment and long-term survival of osteocytes [5,6,7]—the main orchestrators of bone remodeling [8,9]. Selective laser ablation under ambient conditions results in a highly developed TiO_2_ layer (~150–200 nm) on the implant surface, compared to a spontaneously formed TiO_2_ (~4–10 nm), and in addition, a multiscale, combined macro-/micro-/nano- topography mimicking the various length scales of bone architecture [7,10,11,12,13,14].

Rapidly developing 3D printing technologies for metals, e.g., selective laser sintering [15] and electron beam melting [16,17], allow fabrication of implants with a macroporous geometry to achieve bone ingrowth [18,19,20]. A macroporous geometry implies that bespoke, lightweight structures can be built for use in applications that require large implants [21,22]. The technology has also shown great promise in achieving osseointegration of alloys such as cobalt-chromium (CoCr), which are traditionally considered inferior to titanium [23]. In addition to the possibility of tailoring implant geometry, electron beam melting also allows doping of potentially therapeutic elements (e.g., Zr) [24,25].

The biological response to metal/alloy implant surfaces, however, remains strongly governed by the initial events—and particularly by the adsorption of proteins and other constituents of tissue fluid [26], which is a function of characteristics such as surface wettability [27]. It becomes obvious that in the long-term, “implant-driven” biological response, and/or modulation thereof, is restricted by the design parameters. Additional pro-osteogenic components can be added in the form of biomimetic calcium phosphate coatings [28] or biomolecules such as bone morphogenetic protein-2 (BMP-2). Furthermore, synergistic effects can be achieved when combined [29]. Although it has been demonstrated that certain molecular pathways can be modulated, e.g., Wnt signaling pathway through thin hydroxyapatite coatings [30], and that controlled release of therapeutic agents (e.g., anti-osteoporotic drugs) can enhance bone formation in osteopenic conditions [31], many systemic conditions that adversely impact bone quality, or the kinetics of bone formation and/or remodeling cannot be specifically targeted by such methods.

In addition to naturally occurring polymers such as collagen, various synthetic polymers have also been developed and investigated in applications such as guided bone regeneration (GBR) and guided tissue regeneration (GTR). Collagen-based membranes typically demonstrate rapid degradation in vivo [32]. In comparison, synthetic polymers exhibit both resorbable and nonresorbable behavior and can be tailored to achieve the desired characteristics, including degradation kinetics and mechanical properties (e.g., stiffness) [33], however, the pro-osteogenic response remains poor. In some cases, incorporation of biomimetic strategies, e.g., fibrous membranes and addition of mesenchymal stem cells [34], has demonstrated promise.

### 1.2. What Is Epigenetics?

The term epigenetics was first coined in 1942 by Waddington and relates to changes in gene expression that are not encoded in the DNA sequence [35,36]. Epigenetic modifications include chemical alterations of the DNA and its associated proteins, called histones, leading to the remodeling of chromatin and activation or inactivation of a gene. Epigenetic mechanisms are important for cellular reprogramming and for the cell-type specificity [36]. All our cells share the same DNA but mammalians comprise of a diverse repertoire of cell types with epigenetic mechanisms that regulate cell-specific functions and gene expression [37]. However, epigenetic mechanisms can contribute to the development and maintenance of cancer and autoimmune or inflammatory diseases, including periodontitis [38,39]. 

In contrast to the human genome, the epigenome is dynamic and changes in response to environmental factors and during a person’s lifetime. Interestingly, some epigenetic modifications are reversible and can be induced and/or altered by environmental factors, therefore presenting a link between the inherited genome and the environment [38]. In addition, epigenetic mechanisms have therefore been also suggested as potential treatment models for improving individualized drug therapy. Several, so called epidrugs, are currently being tested clinically and some has already been found to potentially improve cancer therapy [39,40]. 

#### 1.2.1. DNA Methylation

Modifications of the DNA do not change the base pairing but affect the DNA–protein interaction while being in the major groove of the double helix [41] DNA methylation is associated with transcriptional repression and hence, silencing of gene expression [42]. Transcriptionally active genes are associated with low levels of DNA methylation.

In the classic DNA methylation model, there is a covalent addition of methyl groups to the 5th carbon on cytosine bases (5-methyl cytosine “5mC”), which are situated next to guanine bases at specific sites in the DNA sequence, so-called CpG islands or CpG sites. The addition of methyl groups is regulated by DNA methyltransferases (DNMTs) [43]. 

To add another level to the concept of DNA methylation, it was discovered that 5mC could be further oxidized into 5-hydroxymethylcytosine (5hmC) by the ten-eleven translocation (TET) family of enzymes [44,45]. The TET enzymes can then further convert 5hmC into unmethylated cytosine, resulting in DNA hypomethylation. The biologic function of 5hmC is not clear yet, but it has been suggested to be an intermediate leading to demethylation of 5mC and thereby re-expression of genes silenced by DNA methylation [46,47].

In 2015, the presence of DNA methylation of adenine, N^6^-methyldeoxyadenosine (6mA), was confirmed in DNA [48,49,50]. Interestingly, a cross talk between methylation of histone H3K4me2 and 6mA was reported [49]. It was hypothesized that this is a trans-generational means of transmitting the epigenetic information. In contrast to 5mC, 6mA presents a broader genomic distribution and is not as tissue specific as 5mC [49]. In bacteria, this epigenetic form plays important roles in DNA replication and repair and host–pathogen interactions as well as in gene expression, but at present, the molecular function of 6mA in eukaryote cells is still unclear [50].

#### 1.2.2. Histone Modifications

Nucleosomes are the building blocks of chromatin [51], and one nucleosome consists of 146 base pair (bp) DNA wrapped around a core histone complex, which includes two copies of each of the following histones: H2A, H2B, H3, and H4. The linker histone H1 connects the nucleosomes, forming the primary chromatin structure, often referred to as ‘‘beads-on-a-string”. Histones can be acetylated or methylated at histone amino acid tails that protrude from the nucleosome [52,53]. Histone acetylation is regulated by histone acetyltransferases (HATs) and histone deacetylases (HDACs). Removal of acetyl groups by HDACs leads to alterations in the packing of DNA around histones and a subsequent inactivation of genes. In contrast, hyperacetylation is associated with transcriptionally active chromatin [54,55].

Histone methylation, in turn, is regulated by histone methyltransferases and demethylases [56]. Histone methylation occurs at lysine, arginine, and histidine, with most studies focusing on the methylation of histones H3 and H4, with H3 lysines K4 and K9 being the most commonly methylated [53,56]. However, other basic residues on histone proteins H1, H2A, H2B, H3, and H4 have been also recently reported [56]. Although high levels of methylated K4 have been associated with transcriptionally active regions, methylated K9 was found in silent chromatin regions [53]. Importantly, a lysine can be mono-, di-, or tri-methylated, further adding to the various functions of methylated histones [53]. However, recent research in the area of histone methylation revealed evidence for an even more complicated regulation and function of histone methylation, with specific methylation patterns in promoters, introns, and exons resulting in fine-tuning of gene expression [56,57].

**Figure 1:**
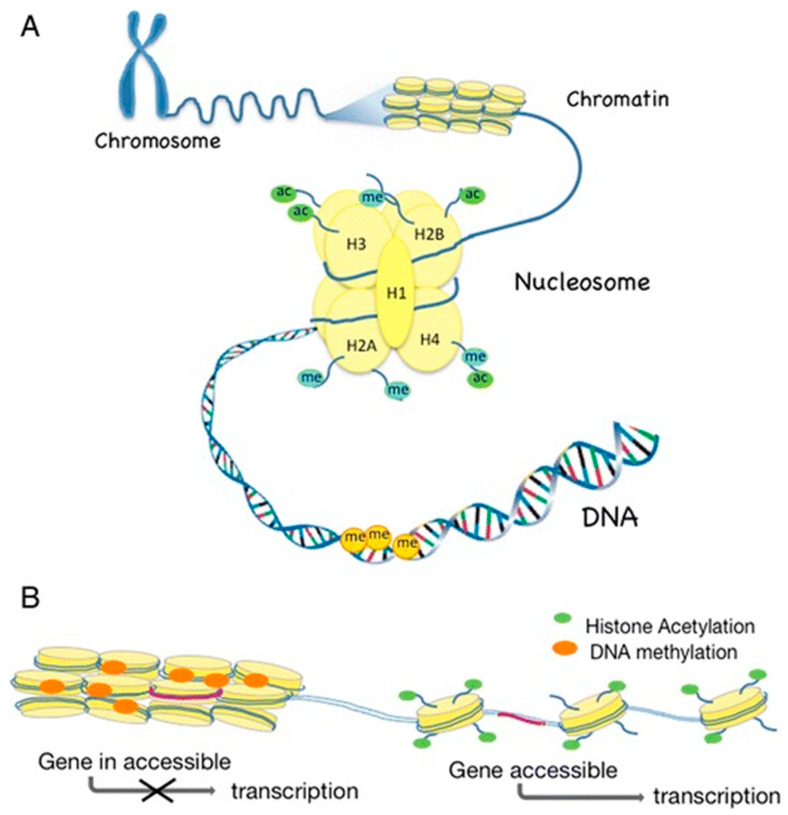
Major epigenetic mechanisms and their influence on gene transcription. (**A**) Chromatin structure (Ac: acetylation; Me: methylation). (**B**) Influence of DNA methylation and histone modifications on chromatin formation and gene expression. From Larsson, Curr Oral Health Rep, 2017. Reproduced under the terms of the Creative Commons Attribution 4.0 International License.

DNA methylation and histone modifications (Figure 1) are not separate events but linked to regulate gene expression and cellular functions [36,58,59].

#### 1.2.3. MicroRNAs (miRNAs)

**Figure 2:**
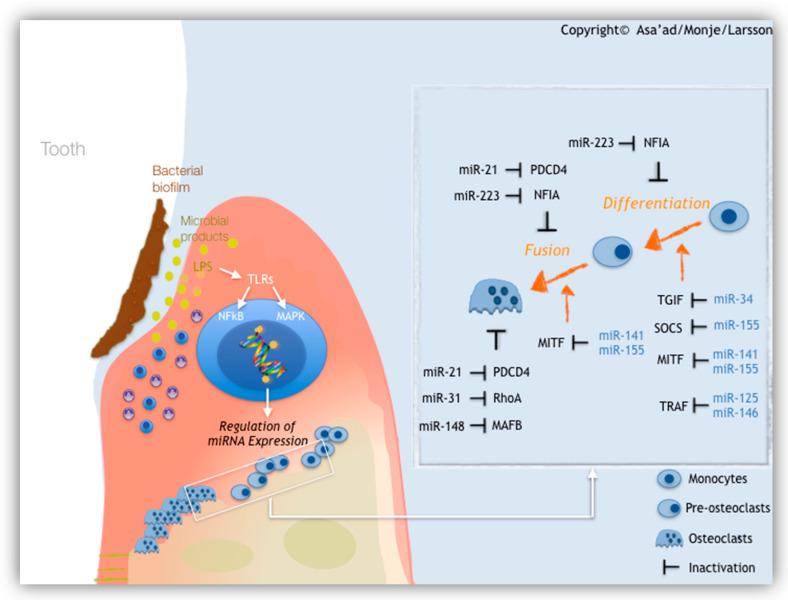
MicroRNAs expressed in periodontitis and their target in alveolar bone. The illustration briefly demonstrates the influence of bacterial biofilm on the expression of miRNAs. When tissues are exposed to bacterial lipopolysaccharide (LPS), expressed miRNAs can increase the sensitivity of toll-like receptors (TLRs) or can target NF-κB signaling pathway or can mediate endotoxin tolerance through modulation of mitogen-activated protein kinase (MAPK) pathway. MiRNAs 21, 31, 148, and 223 are positive regulators of osteoclastogenesis and osteoclastic differentiation. MiRNAs 34, 125, 141, 146, and 155 are negative regulators of osteoclastogenesis and osteoclastic differentiation. From Asa’ad et al., 2019, Eur J Oral Sci. Reproduced with permission from John Wiley and Sons. Copyright 2019.

MicroRNAs (miRNAs) are a group of small noncoding RNAs of about 22 bp in length that regulate gene expression through post-transcriptional modifications of a target messenger RNA (mRNA) [39]. This results in the degradation of a target mRNA or prevention of its translation resulting in suppression of gene expression [60]. Interestingly, one miRNA can control the expression of several genes, while the expression of a certain gene can be controlled by several miRNAs [61]. MicroRNAs are considered as an epigenetic mechanism that modulates cellular processes, such as cell growth, apoptosis, and differentiation, and play fundamental roles in inflammatory responses and the development of diseases, e.g., cancer and rheumatoid arthritis [62]. MicroRNAs have been also suggested as key role players in alveolar bone resorption, contributing to the development and/or progression of periodontitis and peri-implantitis [63,64]. Key microRNAs that participate in alveolar bone destruction due to periodontal disease are illustrated (Figure 2). 

## 2. Materials and Methods

This narrative review examines recently published preclinical studies to address the following focused questions:

(i) Which, if any, are the epigenetic mechanisms used to functionalize implant surfaces to achieve better osseointegration?
(ii) Which, if any, are the epigenetic mechanisms used to functionalize biomaterials to achieve better tissue regeneration?

Electronic and manual searches of the literature in PubMed, MEDLINE, and EMBASE were conducted by two independent reviewers (F.A. and L.L.) for articles published in the last 5 years up to August 2020. The following (or equivalent) search term(s) were used: “(epigenetic OR histone modification OR DNA methylation OR miRNA or microRNA) AND (bone) AND (biomaterial OR implant OR scaffold OR drug delivery) NOT (review) NOT (modular scaffold) NOT (cancer) NOT (CRISPR) NOT (exosome)”.

This search resulted in a total of 185 articles. The exclusion criteria were review papers and papers not in the English language. Two reviewers (F.A. and L.L.) independently extracted the data from studies identified as relevant in the database search. In the following sections, the overall findings are summarized in a narrative manner according to the epigenetically functionalized implant/biomaterial described in the literature retrieved. 

## 3. Epigenetic Functionalization of Implant Surfaces to Enhance Osseointegration

The need for dental implants continues to increase alongside a rapidly growing population [65]. As a result, the field of medical-implant biomaterials has witnessed rapid advancements, particularly in the extensively used titanium and its alloys. Despite these advancements, failure rates of dental implants reportedly range between 3% and 8% [66] due to inadequate osseointegration between the dental implant and host’s bone tissue [67,68]. Therefore, a large number of studies have been dedicated towards discovering new methods of improving osseointegration and reducing the failure rate, with a predominant approach focused on modifying the physiochemical properties of the dental implant’s surface to prompting osteoangiogenesis [69]. The use of epigenetics to coat implant surfaces in order to achieve this goal might be promising. To date, studies in this field have only investigated the potential of miRNAs to coat implant surfaces, without any data on the use of DNA methylation and histone modifications for this specific application. 

In line with this, Meng et al. [70] developed a biodegradable coating, consisting of miRNA-29b encapsulated in nanocapsules in an O-carboxymethyl chitosan coating, to enhance osteogenic bioactivity and was tested in a rat tibial defect model. The authors chose miRNA-29b due to its ability to support osteoblast differentiation and to directly downregulate the inhibitors of osteoblast differentiation, all of which promote osteogenesis [71,72,73], and to decrease the differentiation and function of human osteoclasts [74]. Findings from the in vivo model showed that this coating not only was superior for cell adhesion and growth but also provided sufficient miRNA transfection efficacy and osteoinduction, which significantly enhanced bone regeneration around the titanium surface [70]. 

Another tested miRNA for implant surface coating is miRNA-21, due to its well-documented role in the regulation of cellular functions [75,76,77,78] and its capability in promoting osteogenic differentiation of mesenchymal stem cells [79]. In this context, Geng et al. [80] developed a biodegradable composite coating on titanium implants, made of strontium/hydroxyapatite loaded with miRNA-21 nanocapsules. In vitro results showed that this coating promoted osteoblast proliferation, differentiation, and mineralization, while findings from in vivo implantation in rabbits demonstrated that this coating promoted that expression of the angiogenic factor CD31 and enhanced the expression of osteoblastic genes to facilitate angio-osteogenesis. In addition, the composite coating also showed a decreased RANKL expression. As a result, the composite coating promoted new bone formation and mineralization and thus enhanced osseointegration and bone–implant bonding strength [80].

In another investigation on the use of miRNA-21 for implant surface coating, Wang et al. [81] developed a biocompatible chitosan (CS) and hyaluronic acid (HA) nanoparticles, with the purpose of delivering miRNA-21 (further referred to as CS/HA/miR-21 nanoparticles) into human bone marrow mesenchymal stem cells (hBMMSCs). This was performed by cross-linking the CS/HA/miRNA-21 nanoparticles onto microarc oxidation (MAO)-treated titanium surfaces, in order to establish a miRNA-21 functionalized MAO titanium surface. In vitro results revealed a favorable effect of the implant coating on the osteoblastic differentiation in the hBMMSCs, which was demonstrated by the upregulation of early osteogenesis-related gene markers: *COLI*, *COLIII*, *RUNX-2*, *OPN*, and *OCN*, suggesting that this coating might have a clinical potential in promoting osseointegration [81]. 

It seems that MAO-treated implants are ideal candidates for surface functionalization with miRNAs. Recently, Shao et al. [82] constructed a gene-modified tissue-engineered implant by preparing miRNA-122-modified cell sheets that were complexed into MAO titanium implants. The results of in vitro experiments indicated that miRNA-122 promoted osteogenic differentiation of bone marrow mesenchymal stem cell sheets. 

With the same purpose of modifying the titanium surface, implant surface modification with anti-miRNAs, also known as antago-miRNA, has been explored as well. 

In a recent study, Song et al. [83] used calcium ions (Ca^2+^), in an in vitro model, to deliver siRNA/miRNA to promote osteogenic differentiation of human mesenchymal stem cells (hMSCs) and, thus, improve osseointegration. Since miRNA-138 was previously shown to suppress the focal adhesion kinase signaling, which is essential for osteoblast differentiation, inhibition of miRNA-138 with anti-miRNA-138 might promote the osteogenic differentiation of mesenchymal stem cells [84,85]. Therefore, the authors delivered anti-miRNA-138 into hMSCs from nanotubular titanium implant surface by calcium ions. Their findings showed that Ca^2+^/anti-miRNA-138-functionalized implant was able to significantly enhance mineralization compared to the control groups, both on and around the implant. This finding may be of value for in vivo bone regeneration due to enhanced osteogenesis of hMSCs, both locally and around the functionalized titanium surface [83].

Functionalized titanium surfaces with anti-miRNA-138 were further assessed in vitro and in vivo by Yan et al. [86]. In vivo results revealed a robust vascularized bone formation. The coupling of osteogenesis and angiogenesis observed by this MSC sheet-implant complex could be promising in achieving osseointegration, especially in the compromised bone conditions. 

In an earlier study, Wu et al. [87] evaluated MAO-treated titanium implants, that were functionalized not only with miRNA-29b but also with anti-miRNA-138, in order to enhance osteogenic activity and inhibit the inhibition of endogenous osteogenesis, respectively. Their findings showed that the functionalized surface stimulated the osteogenic differentiation of mesenchymal stem cells as observed by the upregulation of osteogenic expression, enhanced alkaline phosphatase production, collagen secretion, and ECM mineralization. As such, this miRNA functionalized titanium implants can result in a rapid and robust osseointegration at the bone-implant interface. 

In a different investigation, Liu et al. [88] conjugated antago-miRNA-204 with gold nanoparticles (AuNP-antago-miRNA-204) and dispersed them in a poly(lactic-co-glycolic) acid (PLGA) solution, which was applied for coating the surface of titanium implants, by forming an ultrathin sheet on the surface, to promote osseointegration in rats with streptozotocin-induced type 2 diabetes mellitus. The reason behind choosing this specific antago-miRNA was that the authors first identified a highly expressed miRNA-204 in the bone marrow mesenchymal stem cells (BMSCs) of diabetic rats [88]. In the in vitro set-up, the authors reported a successful release of AuNP-antago-miRNA-204 from the PLGA sheet, and taken by the adherent BMSCs, while their results from the in vivo model revealed that this coating strategy indeed has successfully promoted osseointegration in type 2 diabetic rats [88]. Taken altogether, findings of this study suggest that this coating strategy of PLGA sheet/AuNP-antago-miRNA-204 could be a promising strategy in titanium implant surface functionalization, to promote better osseointegration in patients with type 2 diabetes mellitus.

**Figure 3:**
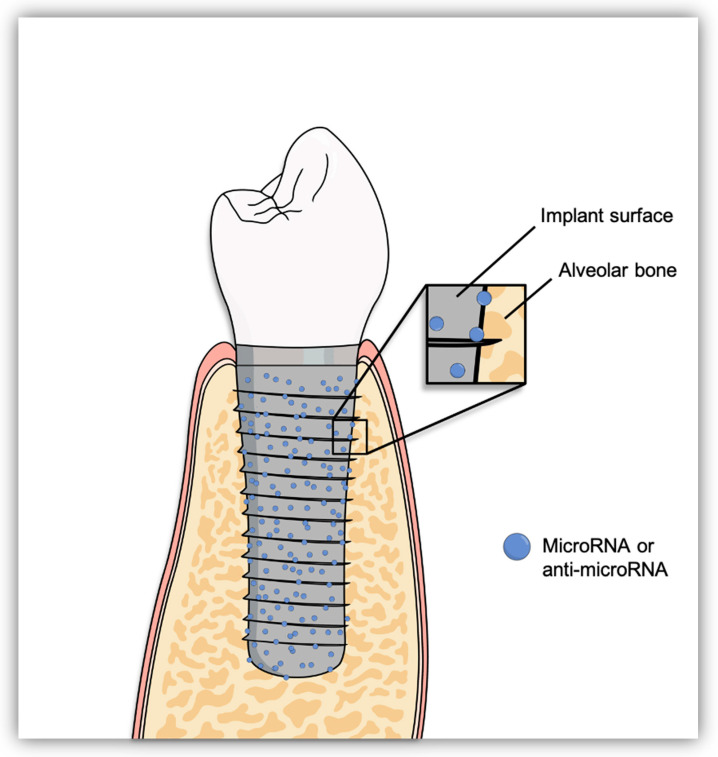
Dental Implant surface coated with microRNA or anti-microRNA to enhance osseointegration.

Taken all the previous findings together, it can be proposed that functionalizing dental implant surfaces with miRNAs or anti-miRNAs might be of importance in promoting osseointegration in healthy and systemically compromised patients (Figure 3). Summary of the previous studies is demonstrated in Table 1.

## 4. Epigenetic Functionalization of Biomaterials to Enhance Bone Regeneration

Controlling the behavior of drug release into the site of injury is important and a challenge in tissue engineering. Thus, the potential to use scaffolds or microspheres as vehicles for site-specific and controlled delivery over time is a major research focus.

Biomaterials for fabrication of scaffolds for bone regeneration can be divided into biodegradable natural polymers including proteins and polysaccharides (collagen, chitosan, and alginate); biodegradable synthetic polymers (polycaprolactone (PCL), polylactic acid (PLA), polyglycolic acid (PGA), and poly(lactic-*co*-glycolic) acid (PLGA); and bioceramics (hydroxyapatite (HAp), tricalcium phosphate (*α*-TCP and *β*-TCP), biphasic calcium phosphate (BCP), and metals (titanium, titanium alloys and magnesium) [89]. For more details about the properties and use of these biomaterials, the reader is referred to some excellent reviews on the topic [89,90]. 

For complex tissues, such as the periodontal tissue with its connection to alveolar bone, composite scaffolds may be used. These composite scaffolds can, e.g., be a polymer combined with ceramics, ceramics/metal, and polymer/metal [89]. 

Regardless of using recombinant protein or genetic material to obtain bone regeneration, these molecules need to be delivered into the healing site. Incorporating growth factors or signaling molecules into a scaffold presents a promising therapy for providing a localized delivery into the healing site. Methods for transfecting a gene into cells include nonviral vectors, viral vectors, and microRNAs [91,92]. In addition, research on using nanoproresolving medicines that not only promote soft and hard tissue regeneration but also reduce the inflammatory infiltrate in the soft tissue is emerging [93].

It has been found that both surface structure and material energy of biomaterials influence cells epigenetic pattern and hence has the ability to further influence gene expression in cells in contact with the material. A recent review summarizing the current knowledge on biomaterials and epigenetics showed that titanium, silica, PLGA, bioglass, and ceramics are potential materials in tissue engineering that influence epigenetic mechanisms [94]. Below, we report findings from studies on the functionalization of biomaterials with epigenetics to enhance bone regeneration and repair. 

### 4.1. Biomaterials and Scaffolds

#### 4.1.1. Natural Polymers

Biodegradable natural polymers such as chitosan and collagen have so far been used together with other materials for the delivery of epigenetic drugs or miRNAs for bone regeneration.

A combination of chitosan, hydroxyapatite, and zirconium dioxide scaffold containing miRNAs for bone tissue regeneration was recently established and shown to have an osteoinductive effect on cells, in fact, the addition of miRNAs further induced the osteogenic differentiation of mouse mesenchymal stem cells [95]. In a study by Wang et al. (2016) [96], osteogenesis in hBMMSCs was accelerated by the delivery of miRNA-21 through chitosan/hyaluronic acid nanoparticles, which resulted in the upregulation of the expression of calcification genes, enhancing alkaline phosphatase production, collagen formation, and mineralized nodule formation [96]. Similarly, Wu et al. [97] utilized chitosan/tripolyphosphate/hyaluronic acid nanoparticles to deliver antimiR-138 to bone marrow mesenchymal stem cells (MSCs). A significant enhancement of the osteogenesis of MSCs was observed by the means of increased expression of osteogenic genes [97]. 

Instead of functionalizing natural polymers solely with miRNAs, the incorporation of 3D-printed technologies to fabricate scaffolds, later to be seeded with miRNA-transfected cells could represent a more advanced approach in this paradigm. In this context, functionalized 3D-printed collagen scaffold with miRNA-148b-transfected bone marrow stem cells were shown to improve clavarial bone regeneration in rats [98]. This innovative approach reflects the applications of 3D printing technologies and also the modification of the biomaterial indirectly by transfecting the seeded cells with miRNAs instead of loading the miRNAs directly into the biomaterial. 

Interestingly, another epigenetic mechanism was investigated in collagen functionalization; collagen sponges and macroporous biphasic calcium phosphate scaffolds mixed with HDAC inhibitor (HDACi) induced woven bone formation and newly formed bone at the contact with the scaffold [99].

Silk is another natural polymer that has been functionalized with epigenetics. James et al. (2019) [100] assessed an anti-sense-miRNA-214 silk device, using surface coating, which not only resulted in a continuous release of miRNA inhibitors up to 7 days in vitro, up to 7 days, but also in the human mesenchymal stem cells, seeded on these devices, expressing higher level of osteogenic genes. These findings could suggest that this novel system could be beneficial for localized bone tissue engineering and in enhancing osteogenesis at the implant surface. 

#### 4.1.2. Synthetic Polymers

Microspheres of PLGA have been used for the delivery of proteins and drugs; by altering its porosity and structure, delivery of drugs and proteins can be released in a controlled way. The microspheres can be porous, nonporous, or covered-porous, with covered microsphere shown to have a sustained release of drug and protein over time compared to open and porous microspheres [101,102]. In this way, by altering the porosity and structure of PLGA, the amount of drug loaded into the microsphere can be controlled, also the release over time, to give the optimum results of the drug at the site of damage or disease.

PLGA microspheres coupled with miRNA-124 coloaded with ketoprofen showed a synergistic effect reducing inflammation as well as bone damage in a rat rheumatoid arthritis (RA) model [103]. Furthermore, the results showed that ketoprofen reduced inflammation in the joints while miRNA-124 reduced cartilage destruction and prevented bone destruction. A sustained release of both agents was present for 3 weeks [103].

Zhang et al. [104] reported on the delivery of miRNA-26a encapsulated into PLGA microspheres immobilized on a nanofibrous cell-free 3D PLLA scaffold, to spatially and temporally control activation of endogenous cells and regenerate critical-sized calvarial bone defects in healthy and osteoporotic mice by targeting *Gsk-3β* to activate the osteoblastic activity of endogenous stem cells. This system can be important in overcoming the limitations of using cell free scaffolds for bone regeneration. This 3D scaffold-defined controlled miRNA delivery technology enables the prolonged expression of multiple osteogenic genes at their therapeutic levels, leading to repair of critical-sized bone defect without adding cells [104].

In an attempt to counteract periodontal bone loss, Liu et al. [105] created a multibiologic delivery vehicle, with a purpose of manipulating T regulatory cells (Treg) cells in situ, since Tregs play an important role in microenvironment modulation for tissue regeneration. This delivery system was made of (a) poly(l-lactic acid) (PLLA) nanofibrous spongy microspheres, as an injectable scaffold for the adhesion and proliferation of Tregs, (b) PLLA/polyethylene glycol (PEG) cofunctionalized mesoporous silica nanoparticles, and c) PLGA microspheres, both utilized to distinctly release IL-2/TGF-β and miR-10a to locally recruit T cells and stimulate their differentiation into Tregs. In a mouse model of periodontitis, the injectable and biomolecule-delivery system resulted in Treg enrichment, expansion, and Treg-mediated immune therapy against bone loss [105].

Xiong et al. [106] used phase separation technology to fabricate a PLLA (poly-l-lactic acid)/POSS (polyhedral oligomeric silsesquioxane) scaffold. MicroRNA-19b-3p-modified BMSCs were seeded on the scaffold, which was then implanted in a critical-sized calvarial defect in rats. The functionalized scaffold promoted efficient healing in the bone defects [106]. 

In order to investigate the use of miRNA as a means to regulate foreign body reaction towards implants, electrospun poly(caprolactone-*co*-ethyl ethylene phosphate) (PCLEEP) fiber scaffold containing miRNAs has been developed [107]. The PCLEEP polymer was suggested to have an enhanced biodegradability compared to PCL. MiRNA-124 and let-7, targeting macrophages and regulating macrophage polarization towards a M2 macrophage phenotype, were used [107]. A sustained release of miRNAs for at least 30 days was reported and the results showed that a scaffold containing Let-7 and miRNA-124 was able to induce a M2 macrophage polarization as well as a thinner fibrous capsule around the scaffold [107]. As a consequence of insertion of an implant specimen, e.g., prosthetics, a foreign body response process starts leading to the formation of a collagenous capsule around the implant specimen. This can influence the function of the implant, especially if the implant is coated with functional molecules. Therefore, the finding of a thinner fibrous capsule may indicate an improved implant integration.

Other various biodegradable synthetic polymers have been functionalized with miRNAs to enhance bone regeneration. Tahmasebi and coworkers (2019) [108] used a bilayer scaffold using electrospinning of poly(ethylene glycol)-*b*-poly(l-lactide-co-ε-caprolactone) (PELCL) forming the inner layer and electrospinning of poly(ε-caprolactone) (PCL) and gelatin forming the outer layer to incorporate miRNA-22 and miRNA-126 to induce osteogenic differentiation of pluripotent stem cells grown on the scaffold [108]. The results obtained from alkaline phosphatase activity, calcium content, bone-related genes, and proteins expression assays demonstrated that the highest osteogenic markers were observed in pluripotent stem cells grown on the miRNA-loaded scaffold [108]. In a calvarial rat defect model, Nguyen et al. [109] tested poly(ethylene glycol) hydrogel seeded with encapsulated hMSCs and miRNA-20a. Their results showed bone formation in the defects, 12 days after postimplantation, reflecting a localized and sustained molecule delivery system [109]. 

Pan et al. [110] utilized a miRNA-29b delivery system using polyethylenimine (PEI)-capped gold nanoparticles (AuNPs) to synergistically promote osteoblastic differentiation. This system exerted a synergistic promontory effect on the osteogenic differentiation of hMSCs and MC3T3-E1 cells, by inducing the expression of osteogenesis genes (*RUNX2, OPN, OCN, and ALP*) [110]. 

#### 4.1.3. Bioceramics

Bioceramic scaffolds are often combined with different polymers in order to improve molecules/drug release from such scaffolds. 

Menciá Castanõ and coworkers [111] designed hydroxyapatite nanoparticles (nHAp) for delivery of miRNA into hMSCs. In addition, they combined the nHAp nanoparticles with collagen to develop a scaffold for bone repair [111]. The same group further investigated the delivery of a miRNA-133a inhibitor to mesenchymal stem cells using the nHAp-collagen scaffold. The results showed an increase in osteogenic differentiation of the cells with an increase in calcium deposition and alkaline phosphatase expression indicating a potential use of this system in bone tissue regeneration [112]. In a different study, nanohydroxyapatite (nHAp) was investigated with polycaprolactone (PCL) to deliver MSCs transfected with anti-miRNA-221 in a calvarial rat defect [113]. The PCL/nHAp nanofibers seeded with MSC transfected anti-miRNA-221 resulted in an enhanced bone healing and increased vascularity [113]. 

Silica has been used for miRNA delivery as well for bone regeneration purposes, especially mesoporous silica nanoparticles, which are ideal for drug delivery and are promising in gene transfection [114]. Recently, Yan et al. [115] encapsulated miRNA-26a mimics into silica mesopores that were coated with PEI. The surface was then conjugated with peptides. This system protected miRNA from degradation and resulted in an increased osteogenic differentiation of rat bone marrow MSCs, even at relatively low concentrations [115].

In an earlier investigation, Lei et al. [116] developed injectable mesoporous silica nanoparticles embedded in poly(ethylene glycol)-*b*-poly(lactic-*co*-glycolic acid)-*b*-poly(*N*-isopropylacrylamide) (PEG-PLGA-PNIPAM) hydrogel for localized and long-term codelivery of microRNA-222 and aspirin, in local mandibular rat defects. The injection of this hydrogel system into the defects resulted in neurogenesis and enhanced bone formation, suggesting that this system could be promising in innervated bone tissue engineering, since adequate innervation is required for an optimal bone healing outcome [116]. 

In the repair of rat femoral defects, Yuan et al. [117] seeded anti-miRNA-26a-5p-modified adipose-derived mesenchymal stem cells (ADSCs) on biphasic calcium phosphate (BCP) scaffolds, which resulted in an accelerated bone formation via the Wnt/Ca^2+^ signaling pathway. This is indeed a very interesting finding, since Yan et al. [115] reported an osteogenic differentiation of rat bone marrow stem cells due to miRNA-26a encapsulated into silica mesopores and Zhang et al. [104] reported on the activation of osteoblastic activity of endogenous stem cells as a result from miRNA-26a loaded into cell-free 3D-printed scaffolds. The discrepancy in findings between the results reported by Yuan et al. [117] and the other two studies might be due to the use of ADSCs in the former. 

To further improve delivery of miRNAs to site of tissue damage and to reduce the risk of degradation of miRNA, Ou and coworkers [118] loaded miRNA-214 inhibitor into a PEI-functionalized graphene oxide (GO) complex. Using this combination, they provided a delivery model with high transfection efficiency as well as controlled sustained release of the miRNA inhibitor. This complex was then assembled into a silk fibroin/hydroxyapatite (SF/HAp) scaffold in order to promote cell adhesion and growth and thus improve bone repair [118]. The results showed an increase in expression of genes involved in osteogenesis.

In another attempt to enhance miRNA delivery and reduce its degradation, Xue et al. [119] developed monodispersed bioactive glass nanoclusters with ultralarge mesopores and tailored nanosize, using branched PEI. This resulted in the protection and delivery of miRNA-5106, which enhanced the osteogenic differentiation of BMSCs, and significantly promoted new bone formation in critical-sized cranial defects in rats. 

## 5. Conclusions and Future Directions

Findings of this review demonstrate the potential of functionalized implant surfaces and epigenetic modifications of biomaterials to better achieve osseointegration and bone formation. Nevertheless, most of these studies explored functionalization of these surfaces through miRNAs. Scarce data are available on the modifications using DNA methylation and histone modification for bone regeneration, despite being commonly used in cancer research. MiRNA and anti-miRNA delivery has been carried out either by directly immobilizing them to the implant surface or by transfecting them in cells and then immobilizing the transfected cells on the surfaces. The composite scaffolds (i.e., more than one biomaterial) have been commonly used in these studies. Further conclusions can be drawn from this review:

- Functionalization of implant surfaces have been done with miRNAs or anti-miRNAs, directly coated on the surface or coated on a biomaterial then attached to the implant surface or by seeding miRNA/anti-miRNA-transfected cells on the implant.
- Unlike cancer studies, which heavily reported on DNA methylation and histone modifications, functionalization of biomaterials and scaffolds for bone regeneration have been done with miRNAs or anti-miRNAs, except for one study that reported on the use HDACis.
- For bone regeneration, the functionalized scaffolds of different biomaterials were either cell free, or were loaded with miRNA or anti-miRNA-transfected stem cells.
- Modulating the inflammatory and immune reaction with these functionalized scaffolds to enhance bone regeneration is possible, either by influencing macrophage polarization or the recruitment of Tregs.
- Microspheres, nanoparticles, and PEI-based nanoparticles are heavily applied for miRNA or anti-miRNA delivery.

### Future Directions

Historically, it has been stated that successful osseointegration of dental implants and biomaterials for bone regeneration is dependent on a direct bone apposition onto the surface of the implanted materials. Recent findings propose that the genetic basis of individuals plays a more critical role than previously described [120]. Previous studies have demonstrated a link between early marginal bone loss around implants and genetic polymorphisms of cytokines such as interleukin (IL-b) [121,122]. Lately, the immune system is believed to play a more decisive role in the biological mechanisms that decide the fate of any biomaterial placed in patients. In many aspects, this means a paradigm shift, where various biomaterials are considered immunomodulatory rather than the more historical view of acting as inert materials. Hence, the focus is currently developing and changing regarding how host molecules and cells interact when reacting to a foreign body with varying chemistry, surface characteristics, and macroscopic design [123,124]. The novel knowledge of the sophisticated mechanisms involved of integration of biomaterials in the tissue brings possible light onto reasons for complications associated with biomaterials insertion. The sensitive chain of reactions involved towards an implanted material runs all the way from initial cellular response down to possible genetic modifications of associated cells.

An interesting hypothesis presented regarding tissue reactions towards various biomaterials is the impact of the basement membrane to which unfavorable transformations could be related. This may lead to alterations in fibroblasts, keratinocytes, collagen, laminin, integrin, and other factors with specific inflammatory and immunologic roles, which eventually lead to foreign body reactions [120]. Hence, epigenetic modifications of biomaterials will probably not only act as the next generation of advanced treatment tools for future regenerative techniques but also bring light in the understanding of specific pathological reactions associated to biomaterials. Although promising preclinical research has been conducted in this area of epigenetic modifications of biomaterials, it remains to be translated clinically. Specifically, most of these preclinical studies focused on functionalized dental implant surfaces with miRNAs, showing promising results in terms of osseointegration. Yet, there are scarce data on the epigenetically modified biomaterials applied in GBR to improve bone regeneration prior to implant placement. Therefore, future research should also focus on studying these biomaterials in terms of alveolar bone regeneration. Furthermore, there is a need to evaluate the efficacy/cost effectiveness of these techniques in comparison to current standards of care in well-designed studies. 

## Figures and Tables

**Table 1:** Summary of studies on functionalized implant surfaces with miRNAs or anti-miRNAs.

Author (Year)	Study Type	MicroRNA and/or Anti-miRNA	Implant Surface Type	Functionalized Coating Method	Results of the Functionalized Coating (FC) Group
Wu et al. (2013) [87]	In vitro	MiRNA 29b and anti-miRNA 138	Microarc oxidation (MAO)-treated titanium surfaces	Lyophilizing miRNA lipoplexes	- FC with miR-29b: Increased expression of *ALP*. Increased expression of *COL1* at day 7. Increased mineralization. - FC with anti-miRNA-138: Increased expression of *BMP*, *OCN*, *OSX*, and *RUNX2.* Increased expression of *COL1* at day 14. Increased mineralization.
Wang et al. (2015) [81]	In vitro	MiRNA-21	Microarc oxidation (MAO)-treated titanium surfaces	Cross-linking of chitosan, hyaluronic acid, and miRNA-21 nano-particles	- Increased expression of *COLI*, *COL3*, *RUNX2*, *OPN*, and *OCN*.
Meng et al. (2016) [70]	In vitro and in vivo (rat tibial defect model)	MiRNA-29b	Machined titanium	MiRNA-29b nanocapsules encapsulated in O-carboxymethyl chitosan coating	- Increased expression of *OCN* and *RUNX2* in vitro. New bone formation was evident in vivo.
Liu et al. (2017) [88]	In vitro and in vivo (diabetic rats)	Anti-miRNA-204	Microarc oxidation (MAO)-treated titanium surfaces	MiRNA-204 conjugated with gold nanoparticles (AuNP-antago-miRNA-204) and dispersed in a poly(lactic-co-glycolic) acid (PLGA) solution	- Increased expression of *BMP*, *OPG*, *ALP*, *RUNX2*, and *COL1* in vitro. High removal torque in vivo.
Geng et al. (2018) [80]	In vitro and in vivo (rabbits)	MiRNA-21	Acid-treated titanium surface	MiRNA-21 nanocapsules encapsulated in strontium/hydroxyapatite coating	- Osteoblast proliferation, differentiation, and mineralization were evident in vitro. - Increased expression of *CD31*, *COL-I*, *RUNX2*, *OCN*, *OPN*, and *OPG* in vivo. - Decreased expression of *RANKL* in vivo.
Shao et al. (2018) [82]	In vitro	MiRNA-122	Microarc oxidation (MAO)-treated titanium surfaces	MiRNA-122-modified cell sheets complexed	- Increased expression of *RUNX2*, *OSX*, *OCN*, *COL1*, *ALP*, and *BMP-2*.
Song et al. (2018) [83]	In vitro	Anti-miRNA-138	Anodized titanium surfaces	Premixed CaCl_2_ and siRNA to form Ca/siRNA coating	- Enhanced osteogenic differentiation of hMSCs on and around the implant surface
Yan et al. (2018) [86]	In vitro and in vivo (mice)	Anti-miRNA-138	Microarc oxidation (MAO)-treated titanium surfaces	Anti-miRNA-138 delivered MSC sheet to the titanium surface, forming MSC sheet–implant complex (MSIC)	- Increased expression of endogenous osteogenesis and angiogenesis-related genes and proteins, alkaline phosphatase activity, extracellular matrix mineralization, and collagen in vitro. - Robust vascularized bone formation in vivo.
